# Exploring the potential prompting role of cervical human papilloma virus detection in vulvar lesions: a cross-sectional study in China

**DOI:** 10.3389/fonc.2024.1353580

**Published:** 2024-02-15

**Authors:** Xiaoqing Dang, Quanlong Lu, Jing Li, Ruifang Li, Bo Feng, Chen Wang, Lifang Gao, Ruimei Feng, Zhilian Wang

**Affiliations:** ^1^ Department of Obstetrics and Gynecology, The Second Hospital of Shanxi Medical University, Taiyuan, China; ^2^ Department of Pathology, The Second Hospital of Shanxi Medical University, Taiyuan, China; ^3^ Department of Epidemiology, School of Public Health, Shanxi Medical University, Taiyuan, China

**Keywords:** vulvar intrepithelial neoplasia, vulva, human papillomavirus, clinical presentation, non-neoplastic epithelial disorders of the vulva

## Abstract

**Introduction:**

The etiology and clinical presentation of vulvar carcinomas, especially vulvar lesions, are not fully understood. Because the vulva and cervix are anatomically connected, human papillomavirus (HPV) is the main cause of cervical lesions. Thus, this study explored the potential characteristics and effects of specific HPV infection types across vulvar lesions and concurrent cervical lesions.

**Methods:**

This retrospective, cross-sectional study analyzed patients with cervical HPV or cytological results and concurrent vulvar biopsy who were seen in our hospital colposcopy clinic in Shanxi Province, China, between 2013 and 2023. Data on age, menopause status, vulvar manifestations, and cytology and HPV infection testing results were collected. Attributable fractions and multinominal logistic models were used to evaluate HPV genotyping and clinical characteristics across vulvar lesions.

**Results:**

Among the 1,027 participants, 83 (8.1%) had vulvar intraepithelial neoplasia (VIN) of high grade or worse (VIN2+), and 127 (12.4%) had non-neoplastic epithelial disorders of the vulva (NNEDV). A total of 175 patients had either VIN2+ or cervical intraepithelial neoplasia (CIN) lesions of grade 2 or worse (CIN2+). The most common HPV genotypes for VIN2+ or concurrent VIN2+/CIN2+ were HPV16, HPV52, and HPV58, although attributable fractions differed among lesions. Patients with normal cytological or histopathological result were more likely to have NNEDV detected, while abnormal cervical diagnosis was associated with higher detection of VIN2+. Multinominal logistic modeling showed that age and HPV16 infection were risk factors for VIN2+ or concurrent VIN2+/CIN2+; however, only vulvar presentation with depigmentation was a risk factor for NNEDV. Among patients with low-grade CIN1/VIN1, compared with those who were HPV16 negative, those who were HPV16 positive were at 6.63-fold higher risk of VIN2+/CIN2+ [95% confidence interval (CI): 3.32, 13.21]. Vulvar depigmentation was also associated with increased risk of NNEDV (odds ratio: 9.98; 95% CI: 3.02, 33.04).

**Conclusions:**

Chinese women may be at specific, high risk for HPV infection types associated with VIN or CIN. The use of cervical cell HPV detection along with vulvar presentation during cervical cancer screening may also contribute to vulvar lesion detection.

## 1 Introduction

Vulvar carcinoma is a rare malignancy, representing approximately 4% of all genital cancers in women (1). According to Global Cancer Statistics 2020, among 185 countries, during that year, there were an estimated 45,240 new vulvar cancer cases (0.2% of new cancer cases) and 17,427 deaths (0.2% of all cancer deaths) (2). Over recent decades, the incidence of vulvar carcinoma has increased, particularly among younger women (3). The most common histology for vulvar carcinoma is squamous cell carcinoma (SCC) (1). Vulvar intraepithelial neoplasia (VIN) and differentiated VIN (dVIN), which originate from squamous cells, are premalignant conditions in vulvar squamous cell carcinoma (VSCC). The etiology of vulvar cancer is largely unknown. To date, human papillomavirus (HPV) infection- and non-HPV infection-related etiological pathways have been summarized. Previous studies found that persistent HPV infection is associated with long-term development of squamous intraepithelial lesion and VSCC. dVIN is more common in vulvar lichen sclerosis (VLS) and, without treatment, is more likely to progress to VSCC than to high-grade vulvar lesions. VLS is a common type of non-neoplastic epithelial disorders of the vulva (NNEDV), which represent a chronic inflammatory disease that may affect any cutaneous site (4), among which the vulvar area is most common.

Neither VIN grades 2 or 3 have an obvious, specific early clinical manifestation and can appear in any vulvar area. The most common NNDEV symptoms are itch and depigmentation, which may be why it is relatively infrequently presented at clinic for further examination and treatment. VIN2/3 are thus often diagnosed late. There also remains no robust screening method. The presence of a thick keratin layer in the vulvar area, which covers the vulvar epithelium, may lead to cytological misdiagnosis. Cytology collection at a spatula end in women with vulvar lesion, after confirmatory biopsy and histopathological examination, reveals only 32% of smears significant for VIN2/3, nor have various other techniques for vulvar cytology testing, including vulvar brush cytology, yielded adequate results, likely because of scarce cellularity. Vulvar cytology testing for VIN2/3 is therefore not currently recommended (5).

The vulva and cervix are anatomical adjacent. HPV testing is effective for cervical cancer screening, and global standards have been developed for cervical sampling and detection methods. Vulvar lesions might thus be detected simultaneous with cervical cancer screening. Identifying HPV infection and its vulvar type would aid a vulvar screening strategy. One meta-analysis suggested that HPV16 and HPV33 are the most predominant HPV genotypes in VIN and that these vary across global regions (6). However, to our knowledge, HPV infection and its vulvar types have been infrequently reported in populations in China, nor are the histological types of various vulvar lesions fully understood, especially in China. Therefore, through a retrospective analysis of a cross-sectional sample of patients in China with different vulvar lesions who were seen over a 10-year period, we aimed to describe the distribution of vulvar lesions. We also explored the potential characteristics and effects of HPV infection type across vulvar lesions and concurrent cervical lesions. The overarching goal was to determine the potential role of cervical HPV in vulvar lesion diagnosis.

## 2 Materials and methods

### 2.1 Study design and participants

This single-center, cross-sectional study was conducted at the Second Hospital of Shanxi Medical University in Shanxi Province, China. All gynecology clinic patients with abnormal cytological results or HPV-positive infection, or abnormal vulvar manifestation, are invited to receive another colposcopy examination. We collected these and other data from the records of all patients who underwent colposcopy-directed vulvar biopsy at the colposcopy outpatient clinic from 1 November 2013 to 31 October 2023. All patients were pathologically diagnosed with vulvar and cervical lesions. Patient age, menopause status, vulvar manifestations, and cytology and HPV infection test results were also recorded by tracking the clinic records system. This study received exemption approval from the ethics committee of the Second Hospital of Shanxi Medical University (2023 YX No. 113).

Patients with vulvar lesions of non-squamous epithelial origin or metastatic vulvar tumor were excluded from analyses. Overall, 1,278 patients underwent vulvar biopsy and were pathologically diagnosed with vulvar lesions during the study period. After excluding patients evaluated more than once within a short interval and those with missing age, menopause status, or lesion information, 1,027 patients were included in analyses.

### 2.2 Cytology and HPV testing

At the gynecological examination, a cervical cytological sample was collected as needed, for cytology diagnosis and HPV testing by the Department of Clinical Laboratory using standard procedures. Cervical cytology was performed by ThinPrep test according to the manufacturer’s protocol (Hologic Medical Technologies Co., Beijing, China; http://www.hologic.com), and results were interpreted and reported by two pathologists based on the 2001 Bethesda System (7).

HPV detection and genotyping were tested using HybriMax HPV Geno-Array kit (Hybribio Biotechnology Limited Corp., Chaozhou, China) with flow-through hybridization and gene-chip methods, according to the manufacturer’s instructions. The HPV Geno-Array can determine 21 HPV types, including 15 high-risk HPV (hrHPV) types (16, 18, 31, 33, 35, 39, 45, 51, 52, 53, 56, 58, 59, 66, and 68) and 6 low-risk HPV types (6, 11, 41, 42, 44, and CP8304) (8, 9).

### 2.3 Vulvar and cervical histological diagnosis

Colposcopy was performed by hospital specialists within 12 weeks, according to a standardized protocol. Vulvar tissue specimens were collected via colposcopy, with all visually abnormal vulva areas biopsied (10, 11). Biopsies were sent to the pathology department, then processed to produce hematoxylin and eosin-stained slides. All slides were evaluated in a blinded manner by two gynecologic pathologists, who were also blinded to the cervical cytological diagnosis and HPV testing results. A third senior pathologist was used to resolve conflicting diagnoses, as needed. Pathological diagnoses for vulvar lesions of squamous epithelial origin included benign inflammatory reaction of the vulva (i.e., vulvitis), low-grade VIN (VIN1), high-grade VIN (VIN2 and VIN3), VSCC, and NNEDV. Pathological diagnoses for cervical lesions included cervical intraepithelial neoplasia (CIN) 1 (CIN1), 2 (CIN2), or 3 (CIN3), and squamous cell carcinoma (SCC).

### 2.4 Statistical analysis

Vulvar lesion distributions across age groups (< 45 years, 45–54 years, 55–64 years, and ≥ 65 years), menopause status (yes or no), vulvar manifestation (none, vulvar itching, depigmentation, masses/vegetations, and/or other symptoms), cervical cytological (NILM or ≥ ASCUS), and histopathological results were categorized. Distribution differences in vulvar lesions among these categories were compared using chi-square or Fisher’s exact probability tests.

Multiple infections required the use of weighting to evaluate the proportions of vulvar or concurrent cervical lesions attributable to specific HPV types (12, 13). Participants with multiple HPV infection types were redistributed when calculating the attributable fraction of a single-type HPV infection. First, the frequency of individual single-type HPV infection across the overall sample was calculated. Then, this proportion was used in weighting of participants with multiple-type HPV infections. For example, for 10 patients who were HPV16 and 18 positive, if there were 40 single-type HPV16 infected and 10 single-type HPV58 infected, then 8 of these 10 multi-type infected participants were HPV16 positive and 2 (10 × 10/[40 + 10]) were HPV18 positive.

Multinominal logistic regressions were used to evaluate the effects of potential risk factors in the incidence of high-grade vulvar or concurrent cervical lesions. Because there was a high correlation (r > 0.5) between HPV and HPV16 infection, and between age and menopause status, variables including age, HPV16 infection, and other factors were included in the models. Statistical analyses were performed using SPSS Statistics for Windows, version 17.0 (SPSS Inc., Chicago, IL, USA). A two-tailed level *p* <0.05 was considered statistically significant.

## 3 Results

### 3.1 HPV infection rate and vulvar and cervical lesion detection rates

**Figure 1:**
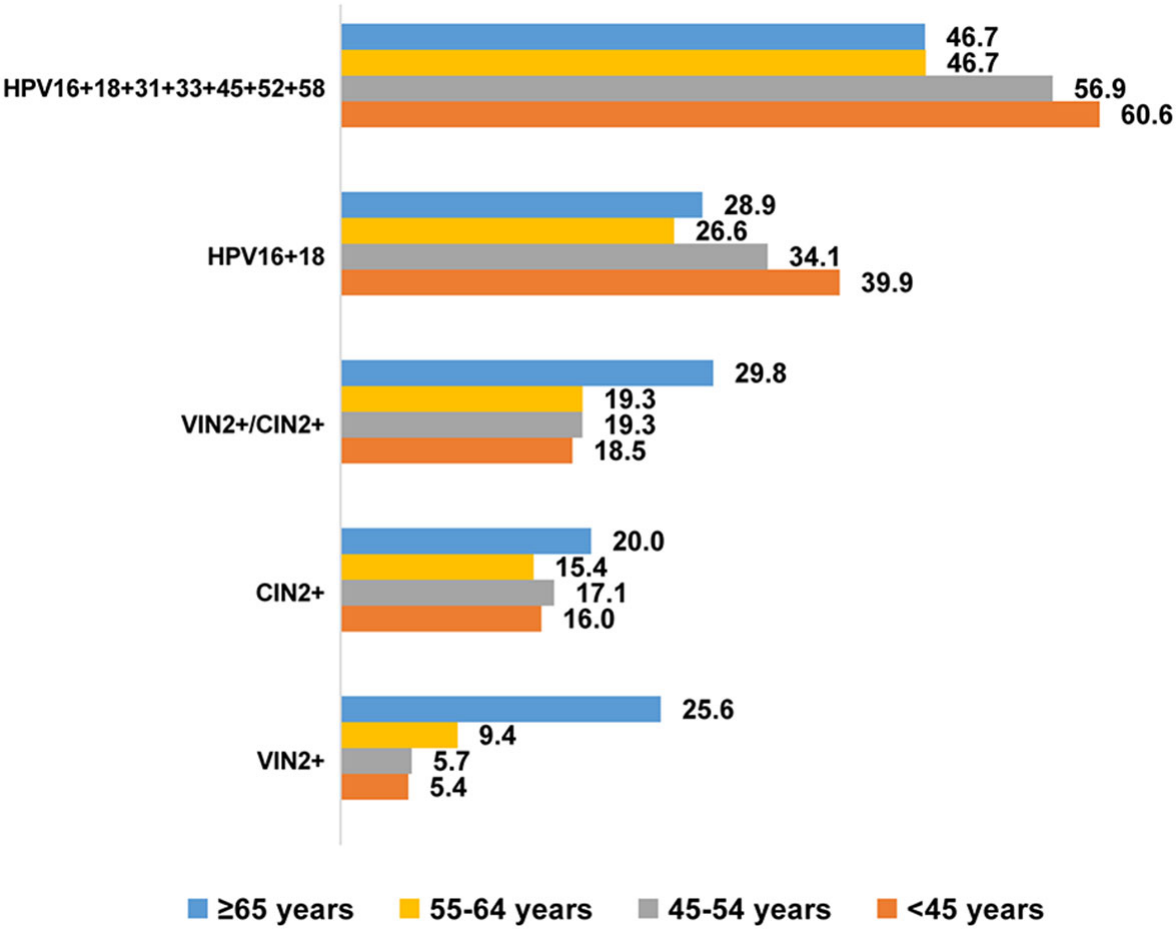
HPV specific-type infection rate (%) and detection rate (%) of vulvar and cervical lesions across different age groups. VIN, vulvar squamous intraepithelial neoplasia; VSCC, vulvar squamous cell carcinoma; VIN2+, vulvar squamous intraepithelial neoplasia 2 and worse; CIN2+, cervical intraepithelial neoplasia 2 and worse.

**Figure 1** shows the specific-type HPV infection rates (%) and detection rates (%) for vulvar and cervical lesions within each age group. Among patients aged ≥65 years, the prevalence of hrHPV infection in nine-valent vaccine (HPV16 + 18 + 31 + 33 + 45 + 52 + 58) was 46.7%, and the prevalence of HPV16 + 18 infection was 28.9%. Among patients aged <45 years, these rates were 60.6% and 39.9%, respectively. Detection rates of high-grade VIN or worse (VIN2+) increased with age, ranging from 5.4% to 25.6% among patients aged <45–≥65 years. There were not significant age group differences in detection rates of CIN lesions grade 2 or worse (CIN2+), which ranged from 15.4% to 20.0%. There were similar rates of concurrent VIN2+ and CIN2+ detection among patients aged ≥65 years (29.8%) and those aged <65 years (18.5–19.3%).

### 3.2 Distribution of vulvar lesions based on patient characteristics

Among the 1,027 patients, 186 (18.1%) were diagnosed with vulvitis, 631 (61.4%) with VIN1, 49 (4.8%) with VIN2/3, 34 (3.3%) with VSCC, and 127 (12.4%) with NNEDV. The average patient age was 47.6 years, and those with VIN2/3 (51.5 years) and VSCC (59.9 years) were older.

**Table 1** shows the distribution of various vulvar lesions, patient characteristics, and clinical features. Within this sample, 58.7% of patients were older than age 45 years. With increasing age, the prevalence of VIN2/3, VSCC, and NNEDV also increased; patients aged <45 years had 3.8% VIN2/3, 1.7% VSCC, and 7.1% NNEDV, while those aged ≥65 years had 10.0% VIN2/3, 15.6% VSCC, and 25.6% NNEDV. A total of 440 (42.8%) participants were post-menopause and had a higher prevalence of high-grade vulvar lesions and NNEDV (6.1% VIN2/3, 5.9% VSCC, and 18.4% NNEDV) compared with patients who were pre-menopausal (3.8% VIN2/3, 1.4% VSCC, and 7.8% NNEDV).

**Table 1:** Distribution of various vulvar lesions among basic characteristics and clinical manifestation.

	vulvitis	VIN1	VIN2/3	VSCC	NNEDV	Overall
	N=186 N (%)	N=631 N (%)	N=49 N (%)	N=34 N (%)	N=127 N (%)	N=1027 N (%)
Age						
Mean (std)	47.9 (11.4)	45.5 (11.6)	51.5 (14.3)	59.9 (14.5)	52.6 (13.0)	47.6 (12.5)
<45 years	71 (16.8)	300 (70.8)	16 (3.8)	7 (1.7)	30 (7.1)	424 (41.3)
45–54 years	61 (20.4)	185 (61.9)	13 (4.4)	4 (1.3)	36 (12.0)	299 (29.1)
55–64 years	42 (19.6)	114 (53.3)	11 (5.1)	9 (4.2)	38 (17.8)	214 (20.8)
≥65 years	12 (13.3)	32 (35.6)	9 (10)	14 (15.6)	23 (25.6)	90 (8.8)
*P* value			<0.001			
Menopause status						
Yes	82 (18.6)	224 (50.9)	27 (6.1)	26 (5.9)	81 (18.4)	440 (42.8)
No	104 (17.7)	407 (69.3)	22 (3.8)	8 (1.4)	46 (7.8)	587 (57.2)
*p*-value			<0.001			
Vulvar manifestation						
None	15 (13.5)	85 (76.6)	6 (5.4)	1 (0.9)	4 (3.6)	111 (10.8)
Itching	12 (29.3)	11 (26.8)	0 (0)	0 (0)	18 (43.9)	41 (4.0)
Depigmentation	14 (25.0)	12 (21.4)	3 (5.4)	1 (1.8)	26 (46.4)	56 (5.5)
Masses/vegetations	16 (12.6)	73 (57.5)	6 (4.7)	22 (17.3)	10 (7.9)	127 (12.4)
Itching and depigmentation	16 (31.4)	14 (27.5)	4 (7.8)	6 (11.8)	11 (21.6)	51 (5.0)
Others	13 (17.3)	14 (18.7)	1 (1.3)	0 (0)	47 (62.7)	75 (7.3)
Unknown	100 (17.7)	422 (74.6)	29 (5.1)	4 (0.7)	11 (1.9)	566 (55.1)
*p*-value*			<0.001			
TCT						
NILM	120 (18.4)	387 (59.4)	27 (4.1)	16 (2.5)	102 (15.6)	652 (63.5)
≥ASC-US	56 (16.5)	238 (70.0)	22 (6.5)	13 (3.8)	11 (3.2)	340 (33.1)
Unknown	10 (28.6)	6 (17.1)	0 (0)	5 (14.3)	14 (40.0)	35 (3.4)
*p*-value			<0.001			
Concurrent cervical lesions						
None	57 (17.4)	142 (43.3)	17 (5.2)	21 (6.4)	91 (27.7)	328 (31.9)
CIN1	91 (17.2)	398 (75.2)	17 (3.2)	3 (0.6)	20 (3.8)	529 (51.5)
CIN2+	27 (19.3)	84 (60.0)	13 (9.3)	6 (4.3)	10 (7.1)	140 (13.6)
Unknown	11 (36.7)	7 (23.3)	2 (6.7)	4 (13.3)	6 (20.0)	30 (2.9)

VIN, vulvar squamous intraepithelial neoplasia; VSCC, vulvar squamous cell carcinoma; NNEDV, non-neoplastic epithelial disorders of the vulva; NILM, negative for intraepithelial lesion or malignancy; ASC-US, atypical squamous cells of undetermined significance; CIN 1, cervical intraepithelial neoplasia1; CIN2+, cervical intraepithelial neoplasia 2 and worse.

*Single itching, single depigmentation, and combined itching and depigmentation were combined into one group.

The most common clinical symptoms and signs within the sample were itching or depigmentation [148 patients (14.5%)], masses/vegetations [127 patients (12.4%)] and other clinical manifestations (7.3%), including vulvar pain, vulvar ulceration, and vulvar atrophy. Patients with vulvar masses/vegetations had the highest prevalence of VIN2/3 (4.7%) and VSCC (17.3%), followed by patients with itching and depigmentation, who had 7.8% VIN2/3 and 11.8% VSCC. The clinical symptoms and signs of 566 patients were unknown; however, most (92.3%) had vulvitis or VIN1.

**Figure 2:**
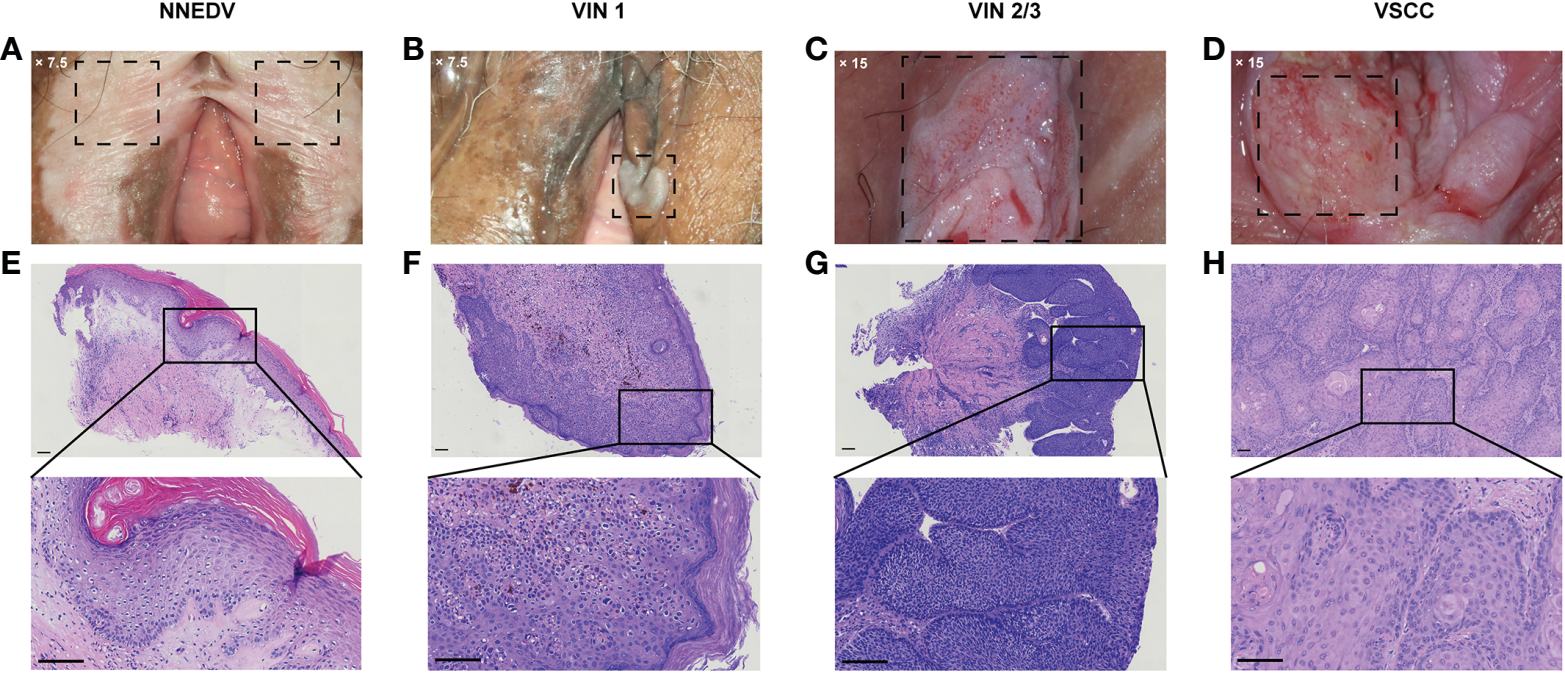
Representative cases of the vulvar lesion. **(A–D)** Images of the typical clinical manifestations of different vulval lesions under colposcopy **(A)** ×7.5; **(B)** ×7.5; **(C)** ×15; **(D)** ×15. (**E–H**) The corresponding hematoxylin–eosin staining pathological images and local magnification maps (magnification: ×100; scale bar: 100 μm). NNEDV, non-neoplastic epithelial disorders of the vulva; VIN, vulvar squamous intraepithelial neoplasia; VSCC, vulvar squamous cell carcinoma.

Among the overall sample, 340 patients (33.1%) had cervical ASC-US or higher, and 140 (13.6%) had concurrent CIN2 or higher. Patients with abnormal cytological results had higher detection rates of VIN2+ compared with patients with normal results (10.3% vs. 6.6%). Similarly, patients with CIN2+ had 13.6% VIN2+; however, patients with normal cervical histopathology also had 11.6% VIN2+. Overall, the distribution of various vulvar lesions differed significantly based on age group, menopause status, clinical symptoms, cervical cytological diagnosis, and cervical histopathological diagnosis. **Figure 2** shows the colposcopic and histopathological findings based on vulvar lesion type.

### 3.3 HPV infection and HPV infection type across vulvar lesion types

**Figure 3:**
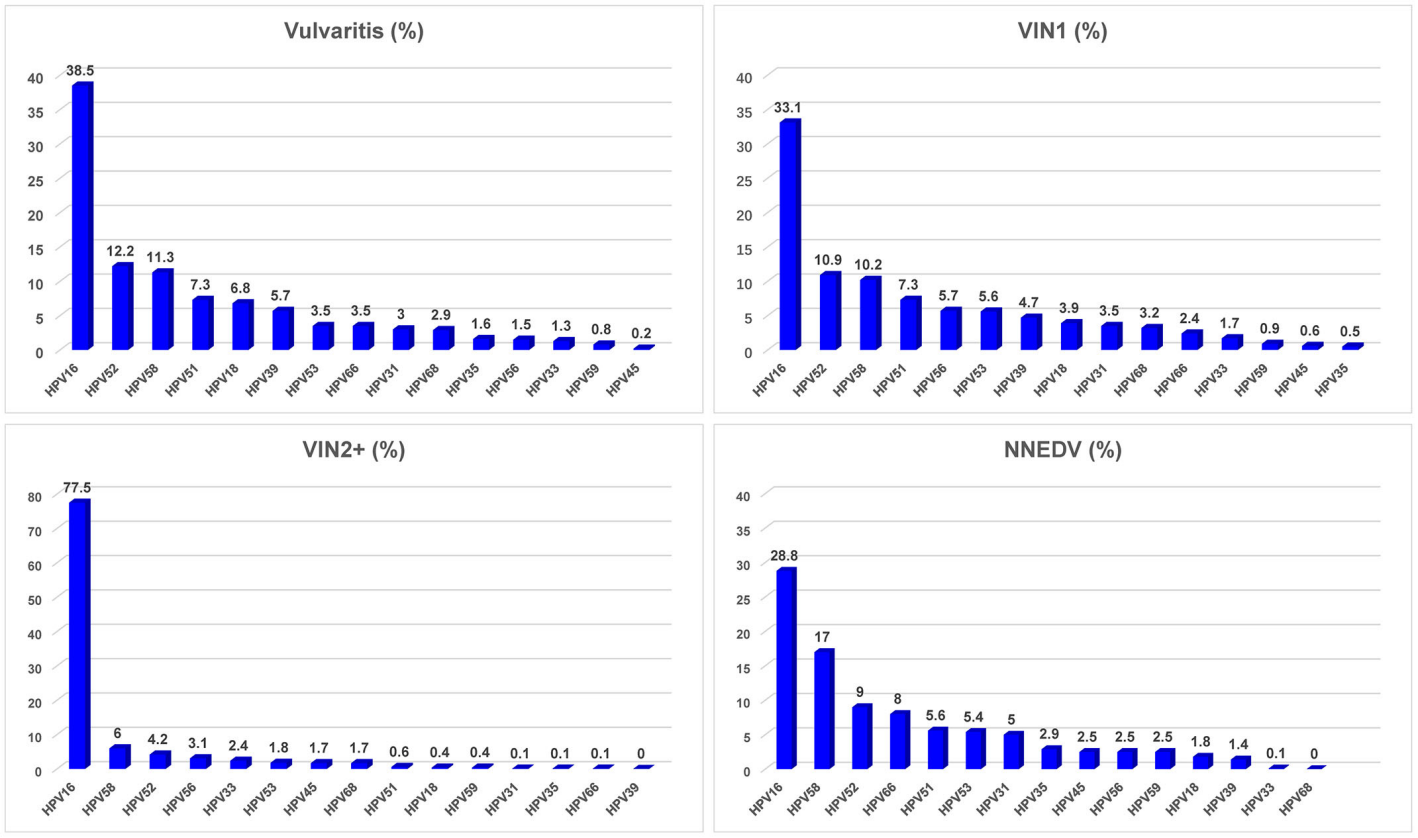
HPV infection rate and distribution of high-risk HPV type among various vulvar lesions. Abbreviations: VIN, vulvar squamous intraepithelial neoplasia; VSCC, vulvar squamous cell carcinoma; NNEDV, non-neoplastic epithelial disorders of the vulva; VIN2+, vulvar squamous intraepithelial neoplasia 2 and worse; hrHPV, high-risk human papillomavirus. *Attributable proportion was calculated using weighting method by distribution of each single HPV type across the overall subjects.

**Figure 3** lists the attributable fractions of various vulvar lesions to HPV infection and specific HPV infection type. There were 768 patients (95.3%) who were hrHPV positive and 38 (4.7%) who were hrHPV-negative; the remaining 221 cases had no HPV testing. Among the patients diagnosed with vulvitis, those with VIN2/3 or VSCC had higher rates of HPV infection than did those with NNEDV. The top 5 most common infection genotypes among the overall sample were HPV16 (36.8%), HPV52 (10.4%), HPV58 (10.3%), HPV51 (6.7%), and HPV53 (4.9%). There was 4%–4.5% of attributable fraction to HPV56, HPV39, and HPV18.

Among patients with vulvitis, the most prevalent hrHPV types were HPV16, HPV52, HPV58, HPV51, and HPV18. Among those with VIN1, similar attributable fractions of the most common hrHPV were observed; fractions for HPV16 were 33%–39%, for HPV52 and HPV58 were ~10%–12% each, and for HPV51 was 7.3%. Among those with VIN2+, the top hrHPV type was HPV16, and its attributable fraction was 77.5%; the other most common hrHPV types were HPV58 (6.0%), HPV52 (4.2%), HPV56 (3.1%), and HPV33 (2.4%). The total attributable fraction of the five hrHPV infections was 93.2%. The five most common hrHPV types among women with NNEDV differed from those with VIN2/3: HPV16 (28.8%), HPV58 (17.0%), HPV52 (9.0%), HPV66 (8.0%), and HPV51 (5.6%). Their total attributable fraction was 68.4%.

### 3.4 HPV infection and HPV infection type across concurrent vulvar and cervical lesions

**Figure 4:**
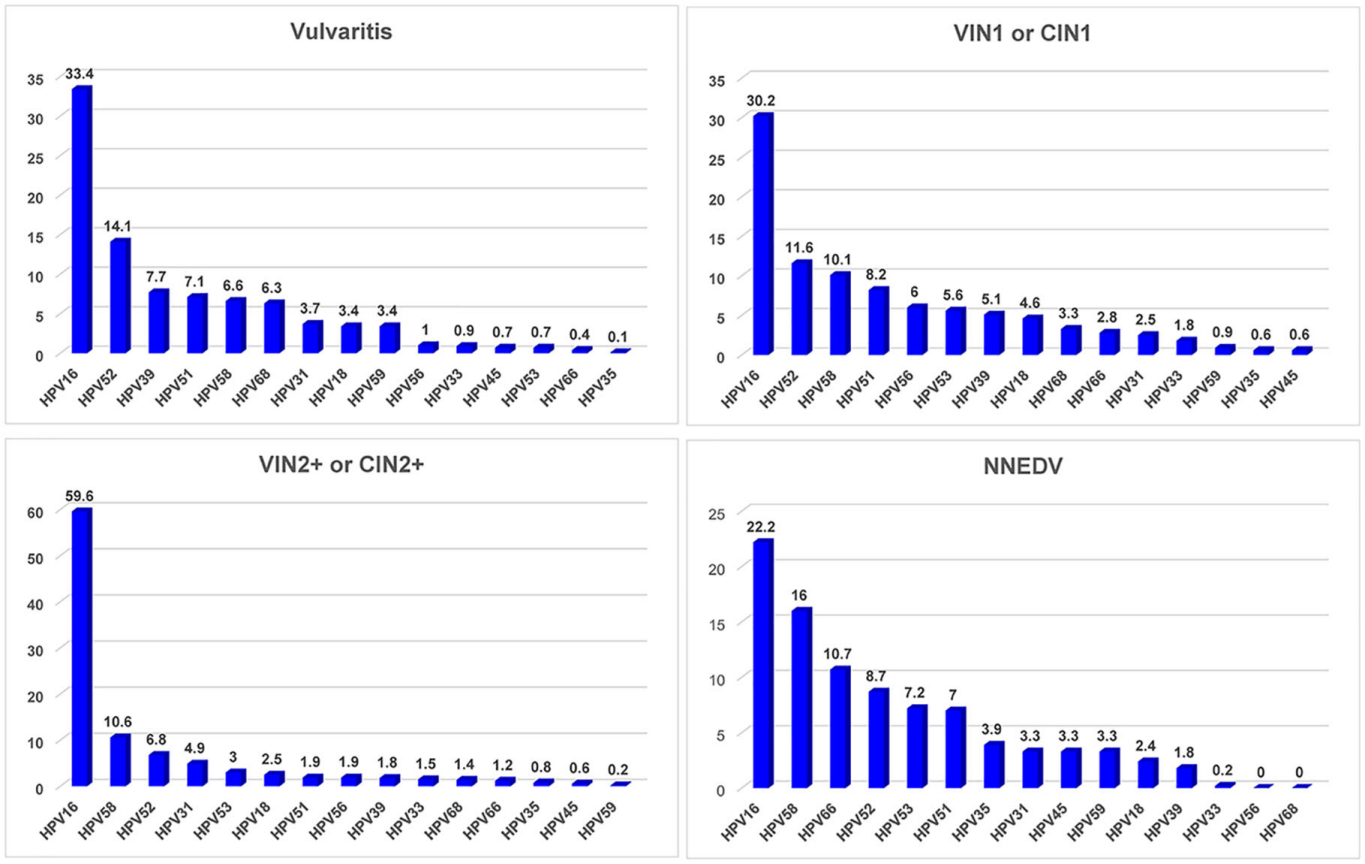
HPV infection rate and distribution of high-risk HPV type among various vulvar and cervical lesions. Abbreviations: VIN, vulvar squamous intraepithelial neoplasia; VSCC, vulvar squamous cell carcinoma; NNEDV, non-neoplastic epithelial disorders of the vulva; VIN2+, vulvar squamous intraepithelial neoplasia 2 and worse; CIN2+, cervical intraepithelial neoplasia 2 and worse; hrHPV, high-risk human papillomavirus. *Attributable proportion was calculated using weighting method by distribution of each single HPV type across the overall subjects.

**Figure 4** presents attributable fractions of concurrent vulvar and cervical lesions to HPV infection and specific HPV infection types. There were 564 patients with combined VIN1/CIN1 and 175 with VIN2+/CIN 2+; 27 had vulvitis and 30 had NNEDV. Patients with NNEDV also had a lower hrHPV infection rate than those with other lesion types.

Among the patients with vulvitis, the top 5 HR-HPV types were HPV16, HPV52, HPV39, HPV51, and HPV58; their attributable proportions were 33.4%, 14.1%, 7.7%, 7.1%, and 6.6%, respectively, and their accumulated attributable fraction was 68.9%. The most common type among different vulvar lesions combined with cervical lesions was HPV16, but its attributable fraction differed. The HPV16-positive rate was 30.2% among patients with VIN1/CIN1, 59.6% among patients with VIN2+/CIN2+, and 22.2% among patients with NNEDV. The four other common hrHPV types differed among various vulvar or cervical lesion types. The accumulated attributable fraction of HPV16/52/58/51/56 was 66.1% among patients with CIN1/VIN1; this value was 84.9% for the attributable fraction of HPV16/58/52/31/53 among those with CIN2+/VIN2+.

### 3.5 Clinical characteristics related to high-grade vulvar lesions or NNEDV

**Table 2** shows the potential risk factors associated with high-grade vulvar lesions and NNEDV. Multinominal logistic model showed that compared with patients with VIN1, those with depigmentation had an increased risk of vulvitis (odds ratio [OR]: 3.79; 95% confidence interval [CI]: 1.26, 11.40) compared with patients without vulvar depigmentation, and a significantly increased risk of NNEDV (OR: 17.63; 95% CI: 5.39, 57.66). Age and HPV16 infection were risk factors for VIN2+. As patient age category increased, the risk of VIN2+ also increased (OR: 2.41; 95% CI: 1.46, 3.98). Patients who were HPV16 positive had an 18.81-fold elevated risk of VIN2+ compared with those who were HPV16-negative (95% CI: 5.77, 61.32). There were no significant associations between vulvar itching or vegetation and VIN2+ or NNEDV risk.

**Table 2:** Risk factors related with high-grade vulvar lesions or NNEDV.

	Cervical or vulvar lesions OR (95% CI)			
	vulvitis N=36	VIN1 N=148	VIN2+ N=27	NNEDV N=29
Risk factors				
Age	1.04 (0.67, 1.63)	Reference	2.41 (1.46, 3.98)	1.52 (0.89, 2.58)
Vulvar itching	2.60 (0.73, 9.23)	Reference	0.46 (0.04, 4.91)	3.59 (0.98, 13.13)
Vulvar depigmentation	3.79 (1.26, 11.40)	Reference	3.35 (0.70, 16.09)	17.63 (5.39, 57.66)
Vulvar vegetation	1.10 (0.47, 2.56)	Reference	2.74 (0.99, 7.58)	1.99 (0.62, 6.47)
HPV16 infection	1.47 (0.65, 3.31)	Reference	18.81 (5.77, 61.32)	1.20 (0.40, 3.60)
	Normal/vulvitis N=10	VIN1/CIN1 N=135	VIN2+/CIN2+ N=65	NNEDV N=25
Risk factors				
Age	1.51 (0.77, 2.93)	Reference	1.69 (1.17, 2.45)	1.63 (0.93, 2.85)
Vulvar itching	1.17 (0.11, 12.45)	Reference	0.39 (0.09, 1.71)	2.45 (0.71, 8.53)
Vulvar depigmentation	3.24 (0.46, 23.13)	Reference	1.62 (0.57, 4.55)	9.98 (3.02, 33.04)
Vulvar vegetation	3.31 (0.73, 14.95)	Reference	0.80 (0.40, 1.64)	1.54 (0.45, 5.22)
HPV16 infection	1.01 (0.20, 5.22)	Reference	6.63 (3.32, 13.21)	1.21 (0.37, 3.99)

VIN, vulvar squamous intraepithelial neoplasia; VSCC, vulvar squamous cell carcinoma; NNEDV, non-neoplastic epithelial disorders of the vulva; NNEVD, non-neoplastic epithelial disorders.

Similar results were found for associations between the above risk factors and risk of concurrent VIN2/3 and CINs. Compared with CIN1/VIN1, those who were older and HPV16 positive had increased risk of VIN2+/CIN2+ (OR: 1.69, 95% CI: 1.17, 2.45; for age; OR: 6.63, 95% CI: 3.32, 13.21 for HPV16 infection), vulvar depigmentation was associated with increased risk of NNEDV (OR: 9.98; 95% CI: 3.02, 33.04).

## 4 Discussion

To the best of our knowledge, our study is the first to describe the distribution of vulvar lesions based on individual clinical characteristics and to evaluate hrHPV infections and their genotypes in patients in China with vulvar and concurrent cervical lesions. Our main findings were that the distributions of vulvar lesions differed significantly by age, menopause status, clinical presentation, and cervical cytology and pathological diagnosis. Vulvar lesions were attributable to the specific hrHPV type, with the five most frequent types being HPV16, HPV58, HPV52, HPV56, and HPV33 among patients with VIN2+. Previous studies have shown that the persistence of HPV infections is one of the most significant predictors for the risk of recurrence of HPV-related cervical and genital lesions (14); therefore, it is important and meaningful to explore the characteristics of HPV infection in vulvar lesions.

### 4.1 Clinical characteristics related to vulvar lesions

Among our retrospective, cross-sectional study, in which patients had vulvar biopsy and pathological diagnosis, we observed detection rates of 4.8% VIN2/3, 3.3% VSCC, and 12.4% NNEDV among women with hrHPV or cytology results. To date, few studies have presented the distribution of vulvar lesion histopathological type, especially including NNEDV. One recent study from Germany reported on 499 women diagnosed with vulvar pathology, showing a similar VLS prevalence (56/499, 11.2%), although it did not report on other lesions (15).

The average age of our patients with VIN2/3 was 51.5 years and 59.9 for those with VSCC. This is consistent with previous research showing that the risk for VIN2/3 and VSCC increases with age (>50 years) (16). Among our patients with NNEDV, 25.6% were older than 65 years. This is consistent with another study showing that with increasing age, the risk of vulvar cancer also increases in women with NNEDV (17). Our study showed that patients who are post-menopause have higher rates of VIN2/3, VSCC, and NNEDV compared with patients who are pre-menopausal. Other prospective studies have shown that women are more likely to develop vulvar cancer after menopause.

Herein, patients with vulvar biopsy were also more likely to have vulvar itching, depigmentation, or masses/vegetations; those with VIN2+ had atypical skin vegetations, and those with NNEDV were more likely to have itching or depigmentation. Several review papers have concluded that the main manifestations of NNEDV are vulvar skin changes and abnormal vulvar sensations; the most common symptom of NNEDV is itching, while VIN2/3 presents with no obvious symptoms. The consistency among other global studies and ours bolsters confidence in these findings. After adjusting for age, multi-presentation (including vulvar itching, depigmentation, and vegetation), and HPV infection, we found that patients with vulvar vegetation were also at elevated risk of VIN2+. Overall, patients with vulvar depigmentation were at an obviously increased risk of NNEDV. NNEDV has garnered increasing attention in recent years because of the high risk of progression to VSCC. Diagnosis of vulvar lesions may be delayed due to the absence of obvious symptoms and routine screening. Therefore, vulvar depigmentation and vegetation could be critical screening signs.

### 4.2 HPV infection and its specific type related to vulvar lesions

Herein, hrHPV infection prevalence was >90%. Among different VIN2/3, women with VIN2+ had 100% hrHPV infection and 77.5% had HPV16 infection. Previous studies have suggested that >80% of vulvar precancerous lesions are HPV positive, while only approximately 25%–40% of VSCC are mediated by HPV-associated pathways (18). A recent meta-analysis reported that low-grade vulvar squamous intraepithelial lesions have a 61.6% pooled prevalence of HPV DNA positivity; the value was 83.3% for the high-grade vulvar squamous intraepithelial lesion, although included studies were highly heterogenous. In Asia, vulvar cancer and VIN2/3 had 48.4% and 82.3% prevalence rates for HPV infection, respectively; the HPV16-positive rate was 66.4% among women with vulvar lesions. In comparison, our study showed a relatively higher hrHPV infection rate than either the global or other regional rates, while our HPV16 infection rate was close to the global level (6). Two possible reasons may partly explain this difference. First, our participants were included based on their HPV test results, among whom the hrHPV infection rate was 95.3%. This may have led to selection bias compared with other studies. Second, different HPV DNA testing methods may alter study results.

We also found that HPV16 was the predominant infection type among VINs and NNEDV, followed by HPV52 and HPV58. Combining across cervical lesions, these three HPV types were also the most frequent overall. Consistent with these findings, another meta-analysis observed that HPV16 (45.7%) was the predominant type, followed by HPV58 (15.5%), HPV52 (11.7%), HPV33 (9.4%), HPV31 (4.3%), and HPV18 (3.5%) among women with cervical lesions (CIN2/3) in China. Another population-based study in China also reported similarly predominant HPV infection types among women with CIN2+ (12). In contrast to our study, that meta-analysis, which included global data, showed that the most common HPV genotype in vulvar cancer was HPV16, followed by HPV33, while in other HPV types predominated by region, HPV18 was the second most frequent type in South America and Asia and HPV52 in Oceania (6). Another study provided similar findings (3). To our knowledge, no previous study has investigated the association between HPV specific-type infection and NNEDV risk, nor are we aware of any other HPV genotyping in vulvar lesions in China. Our study further confirms that Chinese women may have specific hrHPV infection types associated with vulvar or CIN lesions.

### 4.3 Clinical implications

Our finding that, among patients with cervical lesion diagnoses, those with normal cytological or histopathological results had a higher NNEDV detection rate, and that those with abnormal cervical diagnoses had higher VIN2/3 detection rates, suggests that the etiology of VIN2/3 and cervical CINs may be similar and distinct from NNEDV. Several studies have provided similar evidence. For example, there remained an 8% detection of CIN2+ among women treated surgically for high-grade VIN/vulvar cancer but who retained an intact cervix; approximately 25% of these patients with VIN3 had coincident or a history of CIN (19). Women with CIN3 had a 2.68-fold increased risk of vulvar cancer compared with women without CIN3 (OR 2.68; 95% CI: 1.71, 4.18) (20).

Although the etiology of vulvar lesions remains unclear, it is understood to be related to HPV infection with VIN2/3 (21). However, herein, after adjusting for multiple factors, HPV16 infection was not a risk factor for NNEDV compared with VIN2/3; that is, the etiology of VIN2/3 and NNEDV may differ. Potential mechanisms may include that susceptibility to, or opportunistic, HPV infection is increased in one of many ways [e.g., estrogen level changes among post-menopausal women, skin injury, and vulvar area stimulation from itching that causes scratches in patients with VLS (22)], after which it progresses to vulvar cancer. Our study also confirmed that hrHPV is strongly associated with VIN2+ and CIN2+ detection. HPV may thus play an important role in vulvar lesion diagnosis. As such, HPV detection in cervical cells during cervical cancer screenings may likewise aid the detection of VIN2+. We recommend that attention be paid to the vulva during routine cervical examinations and that more education on vulvar presentations and enhanced medical care awareness should be further enforced.

Although this article focuses on the value of HPV in the diagnosis of vulvar diseases, based on the current treatment status of vulvar cancer (23), it is theoretically beneficial to prevent the recurrence of vulvar cancer by clarifying the characteristics of HPV infection and increasing the intervention of HPV during the personalized treatment of HPV-related vulvar cancer. Therefore, it is also meaningful to explore the role of HPV in the treatment and follow-up of HPV-associated vulvar cancer.

### 4.4 Study strengths and limitations

Main study strengths included our concurrent evaluation of hrHPV infection and genotypes for individual histopathological VIN and CIN lesion types, among a single sample, using consistent histopathology, cytology, and HPV genotyping methods, in a population in China. However, the study also had several disadvantages. First, our participants were all seen at the colposcopy outpatient clinic. Most with abnormal cytological results or HPV-positive infection were transferred for colposcopy examination and then further vulvar biopsy and diagnosis. The women with HPV-negative or cytological results were thus not included, likely contributing to a higher HPV infection rate and limiting our ability to evaluate the effects of hrHPV-negative status on risk for vulvar lesions or the prevalence of VIN2/3 among the overall clinic population. Thus, we only evaluated hrHPV infection and attributable fractions of genotypes among various vulvar and cervical lesions. Second, this retrospective, cross-sectional study covered a 10-year period during which gynecologists with different levels of experience were involved in colposcopy examinations and vulvar biopsies, possibly missing some patients with atypical vulvar presentations. While the VIN2+ detection rate herein was 3.3%, reflecting a much higher VSCC incidence (0.2/10,0000) than typical, some cases may have been missed. Finally, we were unable to collect other information on potentially confounding factors, although the focus herein was objective age, menopause status, and cytological and HPV testing results, rather than factors that may influence long-term outcomes.

### 4.5 Conclusion

These retrospective, cross-sectional analyses revealed a NNEDV detection rate twice that of VIN2/3. Our findings suggest that VIN, including VIN1/2/3, and VSCC, compared with NNEDV, have distinct clinical presentations and etiologies. VIN2/3 may be similar to cervical cancer, regarding both etiology and other characteristics. These findings support the notion that greater attention should be paid to the vulva during cervical examination, to increase VIN2/3 detection. Chinese women may have specific hrHPV infection types that are associated with VIN or CIN. The use of HPV detection with cervical cells during cervical cancer screening may also contribute to detection of vulva VIN2+.

## Data availability statement

The original contributions presented in the study are included in the article/Supplementary Material. Further inquiries can be directed to the corresponding authors.

## Ethics statement

The studies involving humans were approved by the ethics committee of the Second Hospital of Shanxi Medical University. The studies were conducted in accordance with the local legislation and institutional requirements. Written informed consent for participation was not required from the participants or the participants’ legal guardians/next of kin in accordance with the national legislation and institutional requirements.

## Author contributions

XD: Data curation, Writing – original draft. QL: Conceptualization, Data curation, Formal Analysis, Investigation, Methodology, Writing – original draft. JL: Data curation, Validation, Writing – original draft. RL: Data curation, Validation, Writing – original draft. BF: Data curation, Validation, Writing – original draft. CW: Data curation, Validation, Writing – original draft. LG: Data curation, Validation, Writing – original draft. RF: Conceptualization, Data curation, Formal Analysis, Methodology, Writing – review & editing. ZW: Conceptualization, Data curation, Formal Analysis, Investigation, Methodology, Writing – original draft, Writing – review & editing.
